# Tablet computer enhanced training improves internal medicine exam performance

**DOI:** 10.1371/journal.pone.0172827

**Published:** 2017-04-03

**Authors:** Daniel C. Baumgart, Ilja Wende, Ulrike Grittner

**Affiliations:** 1 Department of Gastroenterology and Hepatology, Charité Medical School, Humboldt-University of Berlin, Berlin, Germany; 2 Department for Biostatistics and Clinical Epidemiology, Charité Medical School, Humboldt-University of Berlin, Berlin, Germany; Kyoto University, JAPAN

## Abstract

**Background:**

Traditional teaching concepts in medical education do not take full advantage of current information technology. We aimed to objectively determine the impact of Tablet PC enhanced training on learning experience and MKSAP^®^ (medical knowledge self-assessment program) exam performance.

**Methods:**

In this single center, prospective, controlled study final year medical students and medical residents doing an inpatient service rotation were alternatingly assigned to either the active test (Tablet PC with custom multimedia education software package) or traditional education (control) group, respectively. All completed an extensive questionnaire to collect their socio-demographic data, evaluate educational status, computer affinity and skills, problem solving, eLearning knowledge and self-rated medical knowledge. Both groups were MKSAP^®^ tested at the beginning and the end of their rotation. The MKSAP^®^ score at the final exam was the primary endpoint.

**Results:**

Data of 55 (tablet n = 24, controls n = 31) male 36.4%, median age 28 years, 65.5% students, were evaluable. The mean MKSAP^®^ score improved in the tablet PC (score Δ + 8 SD: 11), but not the control group (score Δ- 7, SD: 11), respectively. After adjustment for baseline score and confounders the Tablet PC group showed on average 11% better MKSAP^®^ test results compared to the control group (p<0.001). The most commonly used resources for medical problem solving were journal articles looked up on PubMed or Google^®^, and books.

**Conclusions:**

Our study provides evidence, that tablet computer based integrated training and clinical practice enhances medical education and exam performance. Larger, multicenter trials are required to independently validate our data. Residency and fellowship directors are encouraged to consider adding portable computer devices, multimedia content and introduce blended learning to their respective training programs.

## Introduction

Traditional teaching concepts in medical education do not take full advantage of information technology, despite the fact that modern clinical medicine and biomedical science are packed with digital media resources reaching from multidimensional virtual imaging data of the human body to complex video animations of human physiology.

Medical education ideally happens at the bedside[1], not in lecture halls. Although the use of wireless enabled mobile communication devices[2] including (tablet) computers, personal digital assistants[2] and smartphones[3]–that can help incorporate, process and deliver the ever increasing rich media and information content at the point of care in real time—is substantially increasing[4–6], scientific data on their efficacy in medical education and clinical training is limited.

Here we present prospective data demonstrating that Tablet PC enabled eLearning significantly impacts on exam performance and prospect for future medical trainees.

## Methods

This single center, prospective, controlled study was conducted on an internal medicine ward at Charité Medical Center’s Virchow Hospital, Medical School of the Humboldt-University of Berlin. For the purpose of the study the ward was equipped with three wireless access points (Enterasys, Salem, NH, USA) linking it to the hospital’s intra- and global internet as well as a Net Education Center (Hewlett Packard, Palo Alto, CA, USA), a cart housing and charging Tablet PCs.

### Active participants and controls

Participation was voluntary and in accordance with both institutional policies and all applicable laws including data privacy legislation. The institutional review board of Charité University Hospital in Berlin confirmed the information provided to participants was in line with the local ethical requirements (No.: EA1/386/16). Eligible participants included consenting medical students in their final year of medical school (acting interns) and postgraduate year 1 to 3 residents doing a rotation on the selected internal medicine ward as a mandatory part of their training curriculum. All participants signed a contract consenting to and detailing the conditions of the study. Timing and duration of their rotation were predetermined by medical school, hospital and physician board rules and regulations. Final year medical students (acting interns) interns did four month rotations, while residents did 6 month rotations. The consecutive cohort of all participants was alternatingly assigned to either the active test (tablet) or traditional education (control) group, respectively.

The active test group was profiled and examined (see below), received a Tablet PC to keep for the entire duration of their rotation and use the multimedia training and education package (see below) in- and outside the medical center campus (i.e. at home and commuting to work).

The control group did not receive a Tablet PC and was only profiled and examined (see below) and had access to all conventional education and training resources (i.e. library, books, journals) on campus.

### Objectives and outcomes

The primary objective was to test the hypothesis that Tablet PC enhanced education significantly impacts on participants’ performance in medical board exams. The final MKSAP^®^ exam score was the primary endpoint. Moreover, we aimed to identify participant’s characteristics impacting on the final exam score.

### Participant profiling

Both control and tablet group participants had to complete an extensive questionnaire to collect their demographic data and evaluate their educational status, computer affinity and skills, problem solving strategy, eLearning knowledge and judge their self-estimated medical knowledge to assess potential confounding factors on the overall outcome, respectively.

### Tablet computers

The HP Compaq tc4200[7] (Hewlett Packard, Palo Alto, CA, USA) and IBM ThinkPad X41[8] (IBM, Armonk, NY, USA) are ultraportable notebooks that also convert into tablets. They incorporate technology to provide wireless connectivity and improved battery performance. Tablet PCs are fully functional personal computers delivering performance and compatibility in an innovative form factor. They offer wide-viewing angle displays on protective glass featuring a digital eraser pen that writes like an actual pen.

### Custom software package and programming

We developed a custom, mostly open source (Open Source Initiative, East Palo Alto, CA, USA) software package and named it Mobile Medical Educator (MME). A local Apache (Apache Software Foundation, Delaware, USA) server connected a MySQL database (Oracle, San Francisco, CA, US) with local media content, applications and a graphical user interface (GUI). The GUI was programmed using Java (Oracle, San Francisco, CA, US), CSS and HTML to provide kiosk mode web browser (Firefox, Mozilla Foundation, Mountain View, CA, USA) access for participants to interact with the Tablet PCs.

Through this central interface all participants could register, complete their profile questionnaire, take the initial and final knowledge assessment exams, access a variety of multimedia training and education resources as well as the medical center’s electronic patient care systems.

The American College of Physician℠ (Philadelphia, PA, USA) kindly provided us with a special electronic version (in XML format) of their MKSAP^®^ 14 software that allowed integration into our database system and parsing with a random generator.

The multimedia package included access to the institutional collaborative online course management systems (Moodle and Blackboard), eBooks (Springer Nature Science and Business Media, New York, NY, USA), eJournals, educational slide kits, podcasts, videos, animations, images from major biomedical and scientific publishers or professional societies as well as twitter feeds and selected hyperlinks to biomedical and scientific web resources.

### Initial and final knowledge assessment

To determine the impact of Tablet PC based education we decided to objectively assess and compare all participants’ knowledge in internal medicine at two time points. Importantly, none of the participants had access to or were able to practice the exam questions used in this study or underwent any kind of special knowledge exam preparation.

New medical knowledge recognition[9] and concept identification[10] can be computationally evaluated with the American College of Physicians℠ (ACP) Medical Knowledge Self-Assessment Program MKSAP^®^ first introduced in the 1970s[11, 12]. MKSAP^®^[11] closely resembles the official American Board of Internal Medicine (ABIM) multiple choice question format and style and has been successfully used to evaluate knowledge and analyze currency of ABIM^®^ diplomats[13]. Predictive validity for the ABIM^®^ exam has been demonstrated in the past[14]. The internal medicine exam performance of both, the control and the tablet group was tested by administration of 215 out of 1400 random generator selected, equally distributed questions from all eleven MKSAP^®^ categories (Foundations of Internal Medicine, Cardiovascular Medicine, Gastroenterology and Hepatology, Rheumatology, Neurology, Hematology and Oncology, Infectious Diseases, Pulmonary and Critical Care Medicine, General Internal Medicine, Endocrinology and Metabolism) parsed from the current ACP’s MKSAP^®^ digital edition pool.

Our rationale for using MKSAP^®^ was its proven track record in evaluating internal medicine knowledge. Although primarily designed for resident use, we felt that final year medical students, i.e. acting first year medical residents could be reliably subjected to it as well. In our opinion the benefit of using a vetted, validated questionnaire such as MKSAP^®^ would outweigh its potential limitations and was preferable to designing a brand new knowledge assessment tool.

### Data processing and statistical analysis

All statistical analyses were performed with SPSS 22 (IBM, Armonk, NY, USA) software. For descriptive statistics, means and standard deviations, medians and inter quartile ranges (IQR) or absolute and relative frequencies were reported where applicable. Data are expressed in box plots. Both, the Mann-Whitney-U-test[15] and Fisher’s-exact-test[16] were used to compare participants profile data. The t-test for independent samples or one-way ANOVA was used to test associations of participant’s characteristics with their final score. All variables with a p-value < 0.1 were also tested in a multiple regression model for the final score values. For the multiple regression model self-rated knowledge was dichotomized into excellent/good vs. passable/adequate. Additionally t-tests for related samples were employed to check for significant differences between the mean initial and final exam scores. A two-sided significance level of 0.05 was used. The main hypothesis was the existence of group differences in final scores after accounting for baseline scores and possible confounders. All other tests were secondary. No adjustment for multiple testing was applied.

## Results

### Participant flow and recruitment

**Fig 1:**
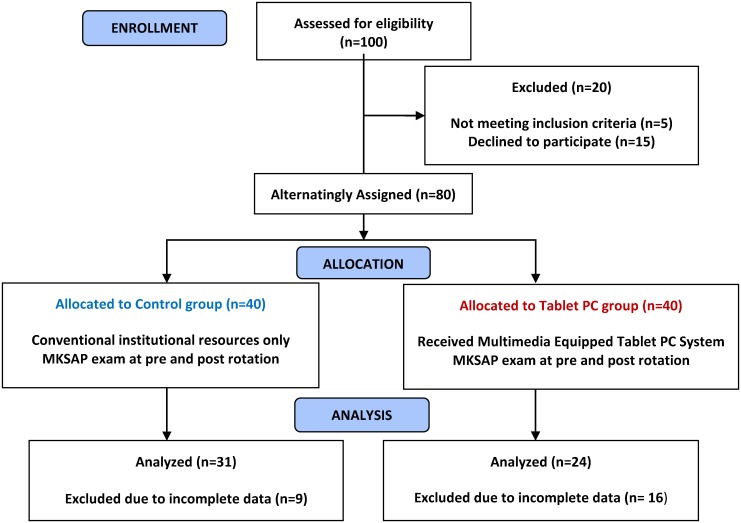
Participant flow.

We recruited 80 participants for this study between 2008 and 2012. Data of 55 participants (tablet n = 24, 50% male; controls, n = 31, 25.8% male; median age 28 years) were evaluable and analyzed. The remaining participants’ data was incomplete and was excluded from the analysis. Fig 1

### Participant profiles

#### Socio-demographics

Most participants were German nationals. There were no statistically significant differences in age, gender or educational background. Table 1

**Table 1:** Participants’ demographics. Parameters were evaluated for statistically significant differences by either Fisher’s exact test or Mann Whitney U test^#^, respectively.

Parameter	Total	Control	Tablet	P Value
	N = 55	N = 31	N = 24	
Gender [n / %]				
• Male	20 / 36.4	8 / 25.8	12 / 50	0.091
• Female	35 / 63.6	23 / 74.2	12 / 50	
Age				
• Median [IQR]	28 (27–29)	28 (27–29)	28 (26–30)	0.742^#^
Nationality [n / %]				
• German	50 / 90.9	29 / 93.5	21 / 87.5	0.643
• other	5 / 9.1	2 / 6.5	3 / 12.5	
Education Level [n / %]				
• Student	36 / 65.5	21 / 67.7	15 / 62.5	0.778
• Resident	19 / 34.5	10 / 32.3	9 / 37.5	
**Exposure to foreign medical education**				
Familiar with USMLE^®^ Exam [n / %]	39 / 70.9	23 / 74.2	16 / 66.7	0.565
Received training in a foreign country [no. / %]	23 / 41.8	14 / 45.2	9 / 37.5	0.595
Received training in the US [n / %]	3 / 5.5	1 / 3.2	2 / 8.3	0.575
**Computer affinity**				
Number of computers owned				
Median [IQR]	1(1–2)	1(1–2)	1(1–2)	0.262#
Current computer type in use [no. / %]				
• Desktop only				0.152
• Notebook/Laptop only	0	0	0	
• Both desktop and notebook/laptop	26 / 47.3	12 / 38.7	14 / 58.3	
	29 / 52.7	19 / 61.3	10 / 41.7	
Portable computer knowledge [no. / %]				
• Tablet PC				
Knows it	28 / 50.9	15 /48.4	13 / 54.2	0.787
Uses it	1 / 1.8	0	1 / 4.2	0.436
• Palm or other PDA usage	4 / 7.3	2 / 6.5	2 / 8.3	0.79
	13 / 23.6	8 / 25.8	5 / 20.8	0.756
Main computer usage location [no. / %]				
• Home				
• Work	53 / 96.4	31 / 100	22 / 91.7	0.186
• Campus	44 / 80.0	25 / 80.6	19 / 79.2	0.892
	13 / 23.6	7 / 22.6	6 / 25.0	0.834
**Current exposure to eLearning**				
Years of eLearning experience Median [IQR]	1.0 (0.5–1.3)	1.0 (0.5–1.0)	0.8 (0.0–1.7)	0.444#
eBook Collection [no. / %]				
• Knows eBook collection	7 / 12.7	3 / 9.7	4 / 16.7	0.686
• Uses eBook collection	11 / 20.0	4 / 12.9	7 / 29.2	0.18
• Subscribed to eBooks	16 / 29.1	10 / 32.3	6 / 25.0	0.765
Resources used for problem solving [no. / %]				
• Article				
• Book catalogue	40 / 72.7	27 / 87.1	13 / 54.2	0.138
• Virtual Library	37 / 67.3	27 / 87.1	10 / 41.7	
• eBook	27 / 49.1	21 / 67.7	6 / 25.0	
• eLearning	12 / 21.8	6 / 19.4	6 / 25.0	
• Other resource	9 / 16.4	7 / 22.6	2 / 8.3	
• No resources	1 / 1.8	1 / 3.2	0	
	11 / 20.0	4 / 12.9	7 / 29.2	
Search Engine used for problem solving [no. / %]				
• Google				
• Pubmed	48 / 87.3	29 / 93.5	19 / 79.2	0.22
• Wikipedia	54 / 98.2	31 / 100	23 / 95.8	0.436
• Other Search Engine	26 / 47.3	14 / 45.2	12 / 50.0	0.789
	17 / 30.9	13 / 41.9	4 / 16.7	0.716
**Self-rated internal medicine knowledge**				
Rating [n / %]				
• Excellent	1 / 1.8	1 / 3.2	0 / 0	0.392
• Good	19 / 34.5	11 / 35.5	8 / 33.3	
• Passable	31 / 56.4	18 / 58.1	13 / 54.2	
• Adequate	4 / 7.3	1 / 3.2	3 / 12.5	
• Failure	0 / 0	0 /0	0 / 0	

#### Exposure to US or other foreign medical education

A fifth of participants had received medical education in foreign countries such as Argentine, Chile, France, Iceland, Italy, Malawi, Russia, Spain, Sweden, Switzerland, The United Kingdom and The United States. However, while many participants were familiar with the term US medical licensing exam (USMLE^®^), only three participants had actually received medical training in the US. None had ever taken the exam. Table 1

#### Computer affinity and skills

Most participants owned at least one computer which was a notebook or laptop in half of the cases. However, they mostly used it at home or work and only in less in quarter of cases in other campus locations. Table 1

#### Currently exposure to eLearning and preferred problem medical solving resources

Participants’ exposure to eLearning prior to this study was very limited with one year of experience on average. Their favorite source for medical problem solving were still articles that they preferably looked up on PubMed or Google, and books. Table 1

#### Self-rated internal medicine knowledge

The majority of participants in both the control and Table PC groups rated their internal medicine knowledge as “passable” or “good” at entry into the study. Only one participant (control group) rated its knowledge as excellent. Table 1

### Outcomes and estimation

#### Improved exam performance in the tablet group

**Fig 2:**
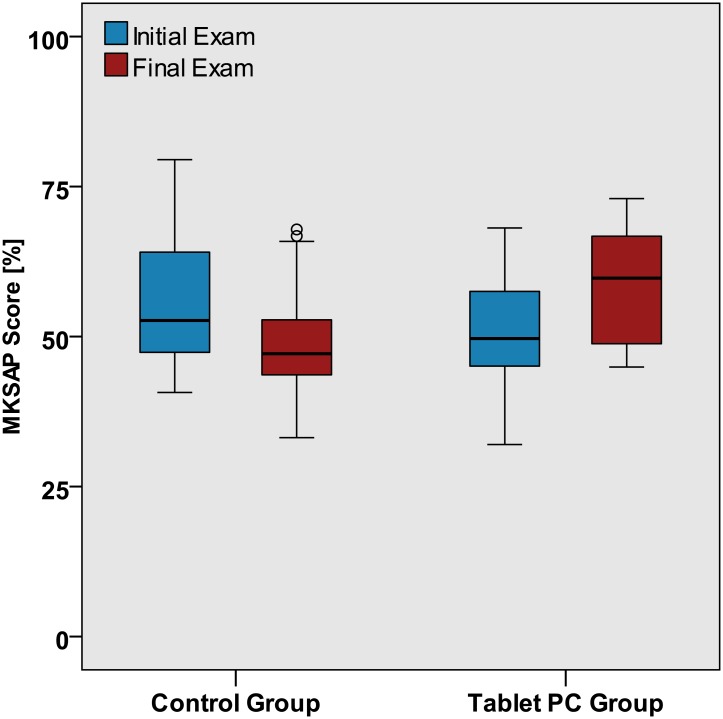
Statistically significant improvement of MKSAP^®^ scores in the tablet but not the control group. Control group (n = 31) mean MKSAP^®^ score Δ — 7 (SD: 11). Tablet group (n = 24) mean MKSAP^®^ score Δ + 8 (SD: 11). The overall result is also reflected in the MKSAP^®^ median initial and final score change distribution by grouped by medical subject categories. Fig 3.

The final mean MKSAP^®^ score was higher in the tablet group (mean (SD): 59 (19)) compared to the control group (mean (SD): 48 (10)) (p<0.001) Table 2, Fig 2.

**Table 2:** Bivariate analysis to identify characteristics associated with final MKSAP^®^ score.

Parameter	N	Final MKSAP^®^ Score	P
		mean (SD)	
	N = 55		
**Group**			**<0.001**
Control	24	48 (10)	
Tablet	31	59 (10)	
Baseline score			0.117
• < = 50	26	50 (10)	
• 51+	29	55 (12)	
Gender			0.161
• Male	20	56 (12)	
• Female	35	51 (10)	
Age			
• < = 27	19	54 (10)	0.599
• 28	18	53 (13)	
• 29+	18	51 (10)	
Nationality			
• German	50	53 (11)	0.717
• other	5	51 (13)	
Education Level			
• Student	36	53 (12)	0.887
• Resident	19	53 (9)	
**Exposure to foreign medical education**			
Familiar with USMLE^®^ Exam	39	53 (11)	0.633
Not familiar	16	52 (11)	
Received training in a foreign country	23	50 (10)	0.157
Training only in home country	32	55 (11)	
Received training in the US	3	49 (3)	0.085
No training in USA	52	53 (11)	
**Computer affinity**			
Number of computers owned			0.11
• 1	37	51 (11)	
• 2 or 3	18	56 (11)	
Current computer type in use			0.883
• Desktop only	0	-	
• Notebook/Laptop only	26	53 (11)	
• Both desktop and notebook/laptop	29	53 (11)	
Portable computer knowledge			
• Tablet PC knowledge	28	55 (11)	0.083
• No tablet pc knowledge	27	50 (10)	
• Smartphone usage	13	55 (12)	0.515
• No smartphone usage	42	52 (10)	
Main computer usage location			
• Home	53	53 (11)	0.415
• Not home	2	59 (7)	
• Work	44	54 (11)	0.066
• Not at work	11	47 (10)	
• Campus	13	54 (13)	0.735
• Not at campus	42	53 (10)	
**Current exposure to eLearning**			
Years of eLearning experience			
• < 1 year	26	51 (11)	0.262
• 1 year	15	52 (9)	
• > 1 year	14	57 (13)	
eBook use			
• yes	11	51 (9)	0.558
• no	44	53 (11)	
Resources used for problem solving			
• Article	40	52 (10)	0.605
• No article	15	54 (13)	
• Book catalogue	34	52 (11)	0.414
• No book catalogue	21	54 (11)	
• Virtual Library	30	52 (10)	0.358
• No virtual library	25	54 (11)	
• eBook	7	54 (7)	0.656
• No ebook	48	53 (11)	
• eLearning	1	58	—
• No eLearning	54	53 (11)	
• Other resource	4	48 (10)	0.356
• No other	51	53 (11)	
• No resources	15	54 (13)	0.56
• At least one of the above	40	52 (10)	
Search Engine used for problem solving			
• Google	48	53 (11)	0.793
	7	52 (8)	
• Pubmed	54	52 (11)	—
	1	71	
• Wikipedia	26	54 (12)	0.63
	29	52 (10)	
• Other Search Engine	17	51 (12)	0.487
	38	54 (10)	
**Self-rated medical knowledge (internal medicine)**			
• Excellent	1	68	**<0.001**
• Good	19	60 (9)	
• Passable	31	48 (9)	
• Adequate	4	54 (12)	
• Failure	—		

**Fig 3:**
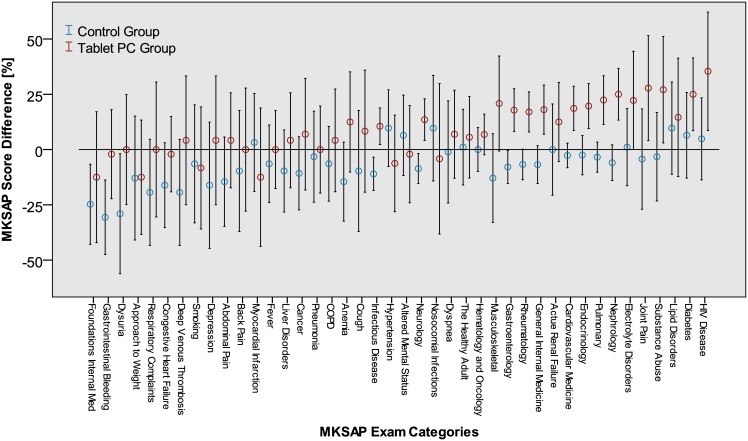
MKSAP^®^ score distribution. Initial and final score change distribution by subject categories in the control (n = 31) and tablet groups (n = 24) Error bars denote 95% CI.

#### Characteristics associated with improved exam performance

At bivariate level baseline of all variables tested only tablet pc use and self-rated excellent internal medicine knowledge at baseline had a significant impact of the final exam score. Table 2 After adjustment for baseline score, tablet pc knowledge and self-rated excellent internal medicine knowledge the tablet group showed on average 11% higher MKSAP test results compared to the control group (p<0.001, main hypothesis) Table 3

**Table 3:** Multiple regression for final MKSAP^®^ score (stepwise variable selection procedure using only significant variables in final model, adjusted for baseline score value), n = 55, R^2^ = 0.58.

	Beta (SE)	p
Intercept	35.6 (6.3)	**<0.001**
Baseline MKSAP score	0.1 (0.1)	0.458
Group (Tablet PC versus Controls)	10.8 (2.0)	**<0.001**
Excellent or good self-rated medical knowledge (internal medicine)	12.5 (2.3)	**<0.001**
Tablet PC knowledge	6.7 (2.0)	**0.001**

## Discussion

We demonstrate for the first time that in a prospective cohort of final year medical students and residents doing an internal medicine inpatient service rotation at an academic medical center the use of a wireless tablet computer based integrated education and portable hospital workstation significantly improves board style exam (MKSAP^®^) performance. This was true even after adjustment for baseline score, Tablet PC knowledge and self-rated excellent internal medicine knowledge.

Unsurprisingly, the overall absolute MKSAP^®^ scores at both time points and in both the control and the tablet group were lower compared with the US national average[17]. This is likely owed to the fact that none of the participants in our study were native English speakers and their exposure to US medical education was very limited. Furthermore, unlike US medical students, residents and foreign (international) medical graduates in the US none had ever taken MKSAP^®^ or ABIM^®^ exams before or participated in regular in-house exams with comparable questions very commonly administered in the US. Moreover, none of the participants practiced MKSAP^®^, USMLE^®^ or ABIM^®^ style exams before or during this study either.

Being naïve regarding this exam type and in relation to prior US medical education can also be considered a strength of our study. Achieving a maximum score was not the goal here, but rather to investigate if the educational tablet system would improve exam performance, i.e. has a significant impact on internal medicine knowledge, which was shown in our results.

Interestingly, the scores significantly worsened in the control group. Perhaps their motivation was lower due to the lack of the incentive of an otherwise desired technical device, which may have been an additional stimulus beyond the actual education software in the tablet computer group. While commonly employed and well proven according to some[18, 19], measurements and metrics may actually also deteriorate individual physician performance[20, 21]. Our study design was unable to detect any such an effect. The difference between Tablet PC and control group could however not be attributed to other socio-demographic factors or computer affinity surrogates either.

Our study furthermore demonstrates that the improved exam performance was significantly associated with the self-rated internal medicine baseline knowledge of participants. This appears plausible as a technical education tool can obviously not replace prior factual medical knowledge acquisition nor supersede basic pedagogic principles.

Our work has limitations. The number of evaluable cases was small and thus the data of this pilot study needs to be validated in larger series before conclusions can be generalized. We also experienced the problem of participant attrition, well known from major educational research studies[22]. The high drop-out rate may relate to the voluntary nature of study participation and perhaps conceiving the extra exams or device as a burden. At the same time the Hawthorne effect [23] also known as observer[24] effect, i.e. the reactivity in which study participants modify or improve aspects of their behavior (exam performance) in response to their awareness of being observed. We have not controlled our analysis for this effect and sample size was likely to small to address this issue. Moreover, only a computer defined random selection of 215 out of all 1400 MKSAP^®^ questions was administered per exam. To avoid skewing of the selection their category distribution was maintained. Still, the MKSAP^®^ edition we used was designed for residents and could have potentially overwhelmed some of the participating final year medical students.

The impact of computer enhanced education on board style exams has been studied before. One group compared scores on preceptor evaluations with National Board of Medical Examiners (NBME) Subject Exam, and a standardized patient (SP)-based exam to complete assigned web cases versus students not completing the assignment. The authors controlled for prior academic performance and clerkship timing using US Medical Licensing Exam (USMLE) Step 1 scores and rotation order. They reported that students completing the web case assignment scored higher on the NBME subject exam and the SP-based exam [25]. Another study examined the impact of a computer-based program where residents receive a score on a Likert-type scale from an attending for each precept based on their knowledge base. The authors found a significant correlation between the resident’s Likert scale scores and their American Board of Family Medicine In-Training Exam scores[26]. Judging from a study in emergency medicine it appears that positive impact of computers is probably independent of the exam style (computerized vs. oral). The authors observed no differences between virtual and traditional groups on critical action scores or scores on eight competency categories[27].

The use of multimedia materials was also studied in dental students, who often have difficulty understanding the importance of basic science classes, such as physiology, for their future careers. The authors reported a significant improvement in unit exam scores[28]

Exam performance without a practical clinical skill level assessment does not automatically translate into superior performance in residency or fellowship programs[29]. The utility of educational games (although our system was not programmed as a game) as a teaching strategy for healthcare professionals remains undetermined according to a recent Cochrane analysis[30]. Moreover, different types of physicians have different needs and preferences for evidence-based resources and handheld devices [31]. Another aspect we could not address in our study was demonstrating the link to improved patient outcomes[32–34].

In summary, our study provides evidence, that tablet computer based integrated training and clinical practice enhances medical education and exam performance. Larger, multicenter trials are required to independently validate our data. Residency and fellowship directors are encouraged to consider adding computer devices, multimedia content and introduce blended learning to their respective training programs.

## Supporting information

S1 File Raw data. This file contains the study raw data, except for any potentially personally identifying information to meet German Federal privacy legislation requirements.
(SAV)
